# The emerging tick-borne pathogen *Neoehrlichia mikurensis*: first French case series and vector epidemiology

**DOI:** 10.1080/22221751.2021.1973347

**Published:** 2021-09-02

**Authors:** Pierre H. Boyer, Lisa Baldinger, Brigitte Degeilh, Xavier Wirth, Chasy Mewa Kamdem, Yves Hansmann, Laurence Zilliox, Nathalie Boulanger, Benoît Jaulhac

**Affiliations:** a Institut de Bactériologie, Fédération de Médecine Translationnelle de Strasbourg, University of Strasbourg, Strasbourg, France; b French National Reference Center for Borrelia, Hôpitaux Universitaires de Strasbourg, Strasbourg, France; c Laboratoire de Parasitologie-Mycologie, Centre Hospitalier Universitaire de Rennes, Rennes Cedex 9, France; d Laboratoire de Parasitologie Médicale Faculté de Médecine, Université de Rennes1., Rennes cedex, France; e Department of Infectious Disease, Strasbourg University Hospital, Strasbourg, France; f Groupe d'Étude de la Borréliose de Lyme (GEBLY), Strasbourg, France

**Keywords:** Tick-borne diseases, post-tick bite fever, *Neoehrlichia mikurensis*, Neoehrlichiosis, vector epidemiology

## Abstract

*Neoehrlichia mikurensis* is an intracellular bacterium transmitted in Europe and Asia by ticks of the *Ixodes ricinus* complex. Interest in this bacterium has increased since it was demonstrated to be responsible for febrile syndromes in patients. To date, most clinical cases have been reported in northern Europe, but case series have also been described in central Europe and China. Notably, thrombotic events occurred during the course of the disease. We investigated the presence of *N. mikurensis* in 10,885 *I. ricinus* nymphs in two regions of France (Alsace and Brittany) collected between 2013 and 2020 and in 934 patients suspected of human granulocytic anaplasmosis in Alsace, an endemic area for Lyme borreliosis, using a specific PCR assay. *N. mikurensis* was detected in 5.42% of the ticks from Alsace, whereas only one (0.03%) tick was found to be positive in Brittany. Spatiotemporal disparities were also noticed within the Alsace region over the four collection sites investigated, and a significant increase in the prevalence of nymphs carrying *N. mikurensis* was also observed in the last three years of collection. Four out of 934 screened patients were found to be positive for *N. mikurensis*. Two had malignancies, and the other two were apparently immunocompetent. Superficial thrombosis was noticed in one patient, and long-lasting bacteremia was noted in another patient. These four patients are the first clinical cases of neoehrlichiosis described in France. We suggest including *N. mikurensis* in the differential diagnosis of post-tick bite febrile syndromes to treat patients and prevent the occurrence of thrombotic complications.

## Introduction

Ixodid ticks are hemophagous arthropods that have a long-lasting blood meal. During this blood meal, they are able to transmit several microorganisms that could lead to diseases in humans [1]. However, although they can host many microorganisms [2,3], a large proportion of these microorganisms have not been identified to be pathogenic for humans yet [4].

Among tick-borne bacteria, interest in *Neoehrlichia mikurensis* has recently increased. This intracellular bacterium was discovered on Mikura Island in Japan in rats and *Ixodes ovatus* ticks [5] but had already been detected in *I. ricinus* ticks and proposed as *Candidatus* Ehrlichia walkerii [6]. Its distribution seems to overlap with that of the two major vectors of Lyme borreliosis in Europe and Asia: *I. ricinus* and *I. persulcatus,* respectively [7]. Moreover, several case reports have recently been reported from northern and central Europe and China [8,9]. Clinically, an infectious syndrome with high fever, arthromyalgias, and chills has been found in immunocompromised patients (especially in patients with B-cell malignancies) as well as in patients without apparent immunosuppression [7,9]. This disease has been designated neoehrlichiosis [7] and could clinically mimic human granulocytic anaplasmosis (HGA). Thromboembolic events, including deep vein thrombosis, have also been reported in immunocompromised patients [10,11], suggesting tropism for endothelial cells. This tropism has recently been confirmed by the cultivation of this bacterium on endothelial cell lines [12]. DNA of *N. mikurensis* has also been detected in humans without apparent symptoms, suggesting that asymptomatic infection could occur [13,14].

Alsace is an endemic region for Lyme borreliosis [15] and *I. ricinus* [16] in eastern France. However, since high fever is not observed among patients with Lyme borreliosis in Europe [17], other tick-borne diseases should be considered in patients displaying high fever after a tick bite, mainly tick-borne encephalitis and HGA.

We, therefore, investigated the presence of *N. mikurensis* in patients suspected of human granulocytic anaplasmosis by PCR. We also evaluated the prevalence of this bacterium in the *I. ricinus* tick vector from the same region as well as in two sites from another region located in western France (Brittany).

## Material and methods

### Study area and ticks collection sites

Patients enrolled in this study were from the north-eastern part of France in the Alsace region, bordering Germany from 2010 to 2019.

**Figure 1.**
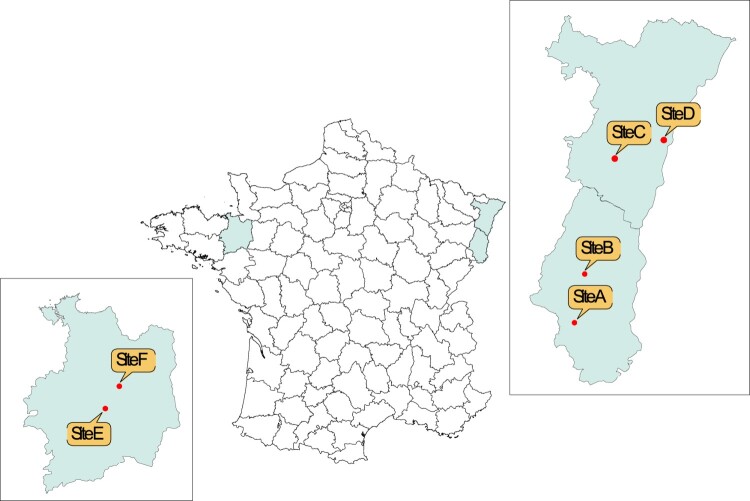
Map of the different collection sites over France, Additional File 1: Analytical performances and characteristics of *N. mikurensis* qPCR, Additional File 2 Table 1: Proportion and number (n) of nymphs carrying *N. mikurensis* and the 95% confidence interval (95% CI) among the collected nymphs (N) at the four sites during the six years of collection. NT: not tested.

Ticks were collected in four sites in the Alsace region. These sites were previously investigated in different studies [16,18,19] and were selected because they display various types of vegetation, environments and ecosystems (i.e. natural or suburban). Sampling site (A) (GPS: 47°42’03.0"N, 7°08’31.0"E) in the Dannemarie Canton where Lyme borreliosis incidence is low, is a lowland forest; sampling site (B) (GPS: 47°55’08.3"N, 7°12’37.6"E) in Murbach area is a mountainous forest with high human activity where Lyme borreliosis incidence is high; sampling site (C) (GPS: 48°26’9.503’’ N, 7°24’42,181"E) near Niedermunster is a mountainous forest; sampling site (D) (GPS: 48°31’07.1"N, 7°44’36.0"E) is a peri-urban forest near Illkirch-Graffenstaden in Strasbourg suburbs (Figure 1).

In order to assess the distribution of *N. mikurensis* in different ecosystems with oceanic climate, two other sites located in the west part of France (in Brittany) were investigated between 2015 and 2020. Sampling site (E) (GPS: 48°05’29’’N, 1°37’25’’W) in Bois de Soeuvres is a lowland forest; sampling site (F) (GPS 48°12’43’’N, 1°30’36’’W) in Forêt de Rennes is also a lowland forest.

### Ticks

Ticks were monthly collected as soon as the weather conditions allowed it by dragging a white flannel flag (1 × 1 m) over low vegetation as previously reported [18].

To assess the prevalence (also called carriage rate) (expressed as a proportion and its 95% confidence interval) of *N. mikurensis* by *I. ricinus* nymphs at the 4 collected sites and over time, ticks were individually tested. A maximum of 60 ticks by site and month of collection was tested. Over the seven years of collection campaign in Alsace, ticks from six years were selected and retrospectively analysed for this study (2013, 2015, 2017, 2018, 2019 and 2020). For the Brittany region, 2015, 2017, 2018, 2019 and 2020 were investigated.

### Patients

Between May 2010 and August 2019, EDTA blood samples from 934 patients were sent to the clinical microbiology laboratory of the University Hospital of Strasbourg for HGA diagnosis by PCR. HGA was suspected (i) when patients presented fever or another symptom presumably related to a tick bite occurring within a maximum of 4 weeks before the beginning of symptoms – or (ii) when patients presented fever and at least one biological abnormality (thrombocytopenia and/or leucopenia and/or abnormal liver function tests) – or (iii) when patients presented the same biological abnormalities without fever, occurring 4 weeks after a tick bite. At the time of consultation, when the blood sample was taken, patients were seen by infectious diseases specialists and an etiological investigation was conducted to establish a differential diagnosis. The present work was approved by the ethic committee of Strasbourg University (No CE-2020-175).

### DNA extraction

For patients, DNA was automatically extracted from 200 µL of EDTA whole blood using two automated extraction devices. From 2010 to 2018, the QIAcube device (Qiagen, Venlo, Netherlands) according to the manufacturer’s instructions using the Cador Pathogen QIAcube HT Kit. Afterwards, the MagNA Pure 96 instrument (Roche Diagnostics, Mannheim, Germany) was used with the MagNA Pure 96 DNA and viral NA Small Volume Kit. These two techniques allowed the extraction of total nucleic acids, in 100 µL of elution volume.

Ticks’ whole DNA was manually extracted immediately after collection with ammonium hydroxide and heat (2 cycles of 15 min heating at 100°C) according to previously published protocols [20,21].

Both tick and patient DNA extracts were stored at −80°C until reanalysis.

### *N. mikurensis* detection PCR assay

A real-time PCR assay targeting the *groel* gene of *N. mikurensis* adapted from Jahfari et al. [22] was performed on ticks’ and patients’ DNA. Briefly, PCR amplifications were carried out on a LightCycler 480. A 20 µL reaction volume was used with 10 µL of LightCycler 480 Probes Master, 3 µL of sample DNA, 0.2 µM of the forward primer and 0.2 µM of a volume-to-volume mixture of the two reverse primers and 0.1 µM of probe.

The PCR conditions were as follows, an initial denaturation at 95°C for 10 min, followed by 60 cycles of amplification (denaturation at 95°C for 5 s, hybridization/elongation at 60°C for 30 s), followed by a final extension step of 30 s at 40°C. A diluted plasmid containing the target gene was included in each experiment as a positive control. A negative control in which the template DNA was replaced with water was also included in each experiment. For patients, PCR products were sequenced using the sanger method with the primers used for amplification. Analytical performances and characteristics of *N. mikurensis* qPCR are summarized in Additional File 1.

### Statistical analysis

Ticks’ *N. mikurensis* carriage rates were calculated and expressed as percentages with their 95% confidence interval using the binom.test(x,n)$conf.int function of the R software, where x is the number of positive ticks and n the number of tested ticks. Statistical modelling using a generalized linear model was performed to assess *N. mikurensis* prevalence in ticks in these four different locations and over the collection years. Data were analysed using R Studio R version 3.4.0 (2017-04-21, https://cran.r-project.org/).

## Results and discussion

### *N. mikurensis* in *I. ricinus* nymphs

During the years of the sampling campaign, 10,885 nymphs were collected. They were all morphologically identified as *I. ricinus*.

In Alsace, the overall *N. mikurensis* rate of carriage by ticks was 5.42% (CI 95% 4.92–5.94%), detailed results are shown in Additional File 2. Interestingly, only one nymph was found to carry *N. mikurensis* from the western part of France (Brittany region). Moreover, the generalized linear models (Table 1) revealed disparities in *N. mikurensis* prevalence in ticks in the four collection sites and over the six years tested. Indeed, the odds ratio of the *N. mikurensis* rate of carriage was significantly higher at site D (OR =  2.81, *p *< .001) than at reference site A. At sites B and C, the rate of carriage was significantly lower than that at site A (OR_site B _= 0.61 and OR_site C_ = 0.16, *p* <.001). The same distribution within the 4 sites was observed during the collection years. Notably, a higher prevalence of *N. mikurensis* among *I. ricinus* nymphs was found in 2018, 2019 and 2020, with the highest prevalence in 2019, 9.03% (95% CI 7.61–10.6%).

**Table 1.** Multivariate analysis of *N. mikurensis* prevalence among the four collection sites in Alsace (A) and over the six years of the collection campaign (B). The results are expressed using the odds ratio and its *p*-value.

	Odd ratio	*p*-Value
*Collection site*		
Site A	ref	ref
Site B	0.61	.003
Site C	0.16	<.001
Site D	2.81	<.001
*Collection year*		
2013	Ref	ref
2015	1.81	.021
2017	1.56	.132
2018	4.26	<.001
2019	5.23	<.001
2020	4.04	<.001

We chose to investigate only *I. ricinus* nymphs because nymphs of this tick species represent the main risk for biting humans [23]. The prevalence of tick-borne microorganisms observed in nymphs is usually lower than that in adults, which have an additional blood meal.

The overall prevalence (5.42%) of *N. mikurensis* in *I. ricinus* nymphs described in this study, in Alsace, is coherent with the rate of carriage of *N. mikurensis* observed in other studies conducted in France in the same region, in surrounding countries and elsewhere in the Northern Hemisphere [9,24,25]. Moreover, the measured prevalence in ticks is close to the prevalence of *N. mikurensis* observed in countries where neoehrlichiosis has been reported [9]. *N. mikurensis* does not seem to be established in the western part of France in Brittany; indeed, only one nymph was found to carry *N. mikurensis*. Future studies are needed to better appreciate the distribution of *N. mikurensis* in French territory. Including this study, *N. mikurensis* has been searched for in only three studies in the eastern part of France [24,25], an area with a high *I. ricinus* density [18]. As this is the first study that explores other regions of France, larger-scale studies are needed to assess the *N. mikurensis* risk of infection.

Interestingly, the highest prevalence of *N. mikurensis* in ticks was observed at site D reaching 23.5% in 2020 and 25% in 2020. Surprisingly, past epidemiological studies carried out on other tick-borne microorganisms at the same collection sites did not show the same distribution. Indeed, the highest prevalence for *B. burgdorferi* s.l. and *B. miyamotoi* was found in site B [19,26]. Since these last two bacteria seem to share the same reservoir as *N. mikurensis* [27], the same distribution for the three microorganisms between the four sites would have been expected. Indeed, a correlation between reservoir-host abundance and the prevalence of tick-borne pathogens has been demonstrated, especially for *B. burgdorferi* s.l. bacteria [28,29]. It can be hypothesized that another reservoir host is more abundant at site D than at the other sites and remains to be identified in future studies.

This trend to increase over the last three years needs to be confirmed in longer-term vector epidemiology studies. Moreover, drivers for this increase may also be highlighted. Here, again, microorganism prevalence within a year strongly depends on the reservoir host abundance in the years prior to this given year [29]. Indeed, a temporal dynamic can be observed with variations in the rate of ticks carrying a given microorganism with acrophases (i.e*.* a period in a cycle during which the prevalence reaches a peak) and bathyphases (i.e*.* a period in a cycle during which the prevalence is low) over several years [30].

Finally, even if disparities were evidenced between the collection sites, this rate of carriage is also in line with the prevalence of other tick-borne pathogens causing febrile syndromes in the same region: *A. phagocytophilum* (ranging from 0.4% to 1.2%) [16] and *B. miyamotoi* (2.18%) [19]. It is also much higher than the prevalence of the tick-borne encephalitis virus found in *I. ricinus* nymphs in Alsace (0.29%) [31], which causes clinical cases in this region [32]. It is therefore natural to question the involvement of *N. mikurensis* in post-tick bite febrile syndromes.

### Infection with *N. mikurensis* in febrile patients

Among the 934 patients previously analysed for HGA, four were found to be PCR positive for *N. mikurensis*. Sequencing of the PCR products revealed a homology of 100% with *N. mikurensis groEL* sequences on the GeneBank database (e.g. MN151367, MH593876.1 and KU535863.1). The clinical characteristics of the four patients are summarized in Table 2.

**Table 2.** Clinical characteristics of the four patients found to have PCR positive for *N. mikurensis*. (Ct: cycle threshold).

Patient	A	B	C	D
*N. mikurensis* PCR Ct	30.72	25.34	38.47	24.54
Sex	M	F	M	M
Age (years)	54	67	61	66
Immunological status	immunocompetent	immunocompromised	immunocompetent	immunocompromised
Comorbidities	—	chronic lymphoid leukemia	—	follicular lymphoma
Splenectomized	no	yes	no	no
Tick bite notion	yes	yes	yes	no
Time between the tick bite and the onset of symptoms	8	—	—	—
Time between the onset of symptoms and the sampling (days)	16	15	77	—
Fever	yes	yes (especially at night)	yes	yes
Headache	yes	no	no	no
Asthenia	yes	yes	no	no
Arthralgia	—	yes	no	no
Duration of symptoms (weeks)	4	—	11	—
Thrombosis	no	no	no	yes
Doxycycline administration	no	yes	no	no
CRP (mg/L)	—	170	37	—
Other	—	—	increase in serum aminotransferases	—

Patient A was a 54-year-old male (blood sample obtained in 2018) without known immunodepression. He very often hiked in southern Alsace, where he was bitten by ticks. Approximately one week after the tick bite, he reported nonspecific infectious syndrome with fever, headache and asthenia. He consulted two weeks later. At the time of consultation, he was apyretic but he still did not feel well. Blood samples for the *A. phagocytophilum* search (by serology and PCR) were taken, which turned out to be negative. Ten days later, he spontaneously recovered without antibiotic treatment. The DNA extract from the EDTA blood sample was retrospectively analysed and found to be positive by PCR for *N. mikurensis*.

Patient B was a 67-year-old woman (blood sample obtained in 2018) with antecedent chronic lymphocytic leukaemia treated with ibrutinib; notably, she was splenectomized. After she reported recent tick bites, she started to present a nonspecific infectious syndrome with fever, arthromyalgias and asthenia. She had fever peaks (40°C of temperature), especially at night. Two weeks later, she was admitted to an internal medicine ward at the hospital. At admission, she had fever, and she was found to have a high C-reactive protein level (170 mg/L); along with neutrophilia, a blood sample to search for *A. phagocytophilum* by PCR was also taken but was negative. She was empirically treated with ceftriaxone (1 g/24 h) and doxycycline (200 mg/24 h) for seven days. She totally recovered with this treatment regimen. The DNA extract from this EDTA blood sample was retrospectively found to be positive for *N. mikurensis*.

Patient C was a 61-year-old man (blood sample obtained in 2010) without known immunodepression who lived in the countryside where he very often practised outdoor activities. While he was hiking, he was bitten by a tick that he did not notice until 24 h later. Two months later, he had fever that lasted for 2 months. At symptom onset, fever was high (40°C) for 48 h. He consulted 77 days after the beginning of the disease. At the time of consultation, fever had already diminished but still had an elevated CRP level (37 mg/L). Blood samples to search for *A. phagocytophilum* by PCR and serology were taken during the medical consultation, and both of them were negative for *A. phagocytophilum*. He recovered without antibiotics and was in good clinical condition two months later. This blood sample was retrospectively found to be positive for *N. mikurensis*.

Patient D was a 72-year-old man (blood sample obtained in 2014). He had stage 2 follicular lymphoma and had been in remission for one year. He also had cerebral venous thrombosis for which he was treated with a vitamin K antagonist four months before his admission to the hospital. He was admitted for superficial femoral venous thrombosis. During his hospitalization, he presented several febrile peaks and chills without an identified primary infection. Because he had fever and because he was exposed to ticks, a blood sample to search for *A. phagocytophilum* by PCR was taken during his hospitalization and was negative. Broad-spectrum antibiotic therapy (ceftriaxone and metronidazole) was initiated because of the fever. No effective antibiotic therapy against *N. mikurensis* was administered. He finally recovered without effective treatment. The blood sample was retrospectively found to be positive for *N. mikurensis*.

These four patients are the first clinical cases of *N. mikurensis* infection in France. Although this bacterium has been observed in the eastern part of France in ticks [24,25], no studies have been carried out on patients in France. To date, cases of neoehrlichiosis have been reported in northern and central Europe, especially in Sweden [10,33], Norway [34], Germany [35], Switzerland [36], and the Czech Republic [37] and more recently in Austria [38]. Cases have also been reported in China [8].

This cohort of 934 patients represents the largest cohort of post-tick bite febrile patients screened for *N. mikurensis*. Whole EDTA-blood samples were addressed to our laboratory to search for *A. phagocytophilum*. Indeed, these patients presented a nonspecific infectious syndrome with high fever, mainly during the summertime, which could be either human granulocytic anaplasmosis or infection by *N. mikurensis* [7]*,* although *A. phagocytophilum* causes cytopenia [39]. However, this latter infection seems to be more frequent than *N. mikurensis* in human infection. Indeed, a part of this cohort (155 patients) was enrolled for a study on anaplasmosis, and 19 out of 155 patients (14 using PCR and 5 using serology only) were diagnosed with anaplasmosis as it has previously been published by *Hansmann et al.* [39], whereas only one of these patients was found to have neoehrlichiosis. In addition, only four out of 934 were found to be positive for *N. mikurensis*, no patient had both *N. mikurensis* and *A. phagocytophilum*. This contrasts with the fact that *N. mikurensis* is at least as prevalent in ticks or even more so than *A. phagocytophilum* [26]. Then, it can be assumed that *N. mikurensis* causes more asymptomatic infection than *A. phagocytophilum*. This is corroborated by the fact that the DNA of this bacterium has also been detected in asymptomatic patients in Poland and in the Netherlands [13,14]. It can also be hypothesized that *N. mikurensis* is currently underdiagnosed since it is not always mentioned by the physician in the presence of an unexplained febrile syndrome in an immunocompromised patient as well as in immunocompetent patients exposed to tick bites.

The clinical pictures and underlying conditions described in our patients perfectly fit with what has already been reported in the medical literature. This bacterium appears to be pathogenic in both immunocompetent and immunocompromised patients [9]. Indeed, two of our patients (patients B and D) had a history of lymphoid malignancies, and one of them was splenectomized (patient B). These comorbidities have repeatedly been reported as risk factors for acquiring infection by *N. mikurensis* [10,36,37,40].

Patient D, who was admitted to the hospital for superficial femoral venous thrombosis and had a history of cerebral venous thrombosis four months before his hospitalization. This indicates that this patient had risk factors for thrombosis, so we can only hypothesize that the infection exacerbated a pre-existing condition. We cannot be certain that neoehrlichiosis was the cause of this thrombosis; nevertheless, thrombosis should be highly monitored in patients with neoehrlichiosis. Indeed, several reports emphasize the fact that thromboembolic events, including deep vein thrombosis, could occur during the course of *N. mikurensis* infection, especially in immunocompromised patients [10,34]. *N. mikurensis* tropism for endothelial cells [12] is probably responsible for the development of thrombosis. To provide adequate monitoring of patients with fever after tick bites, especially those who are immunocompromised, *N. mikurensis* should be specifically searched for, together with *A. phagocytophilum*.

Another interesting observation is the duration of bacteremia, notably in patient C, who remained bacteremic for more than two months (77 days). This fact is corroborated by several studies on *N. mikurensis* in immunocompromised and immunocompetent patients [13,33,34]. This long-lasting bloodstream infection allows the late diagnosis of neoehrlichiosis using molecular tools such as PCR because thus far, there is no reliable serological assay allowing the retrospective diagnosis of *N. mikurensis* infection [41]. Moreover, it was reported to be impossible to differentiate *N. mikurensis* infection from *A. phagocytophilum* infection by serology [41]. Thus, PCR should be performed even long after the onset of symptoms, particularly to provide adequate treatment and monitoring, especially for immunocompromised patients.

## Conclusion

This study provides the first insight into *N. mikurensis* in France. We found this bacterium in the eastern part of France at a significant level in ticks. Increased knowledge of the *N. mikurensis* distribution will help to better prevent the risk of neoehrlichiosis in humans in our country. Spaciotemporal disparities have been highlighted in this study, which should be investigated with research on animal reservoirs in different regions of France to better understand the enzootic cycle of *N. mikurensis*.

Four patients were found to be bacteremic with *N. mikurensis*. The course of their clinical picture and their comorbidities perfectly matched the cases described in the literature.

We propose that *N. mikurensis* should be included in the differential diagnosis of human granulocytic anaplasmosis. To provide better monitoring of thrombotic complications, indications for testing for *N. mikurensis* could also be broadened to unexplained febrile syndromes in immunocompromised patients in endemic areas for tick-borne diseases.

## Supplementary Material

Additional_File_2_Table_1.xlsx

Additional_File_1.docx
