# Supplementary material for: The emerging tick-borne pathogen Neoehrlichia mikurensis: first French case series and vector epidemiology
Source: Emerg Microbes Infect. 2021 Sep 2;10(1):1731–8. doi: 10.1080/22221751.2021.1973347 (PMC8425734; doi:10.1080/22221751.2021.1973347)
Supplement: Additional_File_1.docx [file TEMI_A_1973347_SM5814.docx]

**ADD FILE n°1 : Analytical performances and characteristics of *N. mikurensis* qPCR**

**Background.** This qPCR assay has been adapted from Jahfari et al. (Parasites & vectors, 2012) and used in Jahfari et al. (PLOS Neglected Tropical Diseases, 2016). The authors described a multiplex qPCR for the detection of *A. phagocytophilum* and *N. mikurensis*. Primers and probes targeting the *groEL* gene of *N. mikurensis* were selected to develop a simplex qPCR for *N. mikurensis*. This qPCR assay is designed to be performed on LightCycler® 480 Instrument (Roche).

**Table 1. Primer and probes used for the detection of *N. mikurensis***

| NeoehrF | 5’-CCTTGAAAATATAGCAAGATCAGGTAG-3’ |
| --- | --- |
| NeoehrR | 5’-CCACCACGTAACTTATTTAGCACTAAAG-3’ |
| NeoehrR2 | 5’-CCACCACGCAACTTATTTAGTACTAAAG-3’ |
| NeoehrProbe | (6FAM) 5’-CTAATTATTGCTGAAGATGTAGAAGGTGAAGC-3’ (BHQ1) |

A second reverse primer was designed to cover the diversity of *N. mikurensis* sequences described the Genebank database. Indeed 3 sequences in Genebank carried the mutation C > T in position 21 in the reverse primer (KU865477.1, JQ359067.1 and AB074461.1).

**Table 2. PCR mix**

| Template DNA | 3 µL |
| --- | --- |
| LightCycler 480 Probes Master | 10 µL |
| NeoehrF | 0.2 µM |
| NeoehrR + NeoehrR2 | 0.2 µM |
| NeoehrProbe | - 1. µM |

**PCR conditions :** LightCycler® 480 Instrument (Roche):

- initial denaturation at 95 °C for 10 min
- 60 cycles of amplification
  - denaturation at 95 °C for 5 seconds
  - hybridization/elongation at 60 °C for 30 seconds
- final extension step of 30 seconds at 40 °C

**Specificity.** To assess specificity a panel of 84 control DNA of clinically relevant microorganisms and/or arthropod bacteria (including bacteria closely related to *N. mikurensis*) and DNA of HLA cells (Table 3) were tested with the qPCR assay. None of them presented an amplification curve.

**Table 3.** Specificity panel tested

| *Acholeplasma laidlawii* | *Ehrlichia* spp. | *Mycoplasma orale* |
| --- | --- | --- |
| *Acinetobacter baummanni* | *Eikenella corrodens* | *Mycoplasma pneumoniae* |
| *Actinomyces neuii* | *Enterococcus faecalis* | *Mycoplasma salivarium* |
| *Actinomyces odontolyticus* | *Erysipelothrix rhusiopathiae* | *Neisseria gonorrhoeae* |
| *Aeromonas hydrophila* | *Escherechia coli* | *Neisseria meningitidis* |
| *Anaplasma phagocytophilum* | *Flavobacterium* spp | *Nocardia nova* |
| *Arthrobacter spp* | *Francisella tularensis* | *Nocardia spp* |
| *Bacteroides fragilis* | *Fusobacterium* spp. | *Peptostreptococcus anaerobius* |
| *Bartonella alsatica* | *Gardnerella vaginalis* | *Prevotella melaninogenica* |
| *Bartonella bacilliformis* | *Haemophilus influenzae* | *Proteus mirabilis* |
| *Bartonella henselae* | HLA cells | *Pseudomonas aeruginosa* |
| *Borrelia afzelii* | *Klebsiella aerogenes* | *Rhodococcus equi* |
| *Borrelia burgdorferi* ss. | *Klebsiella pneumoniae* | *Rickettsia conorii* |
| *Borrelia garinii* | *Legionella pneumophilia* | *Rickettsia helvetica* |
| *Borrelia valaisiana* | *Leptospira interrogans* | *Rothia dentocariosa* |
| *Brucella melitensis* | *Listeria monocytogenes* | *Salmonella typhimurium* |
| *Burkholderia cepacia* | *Micrococcus* spp | *Serratia marcescens* |
| *Campylobacter jejuni* | *Moraxella catarrhalis* | *Staphylococcus aureus* |
| *Capnocytophaga gingivalis* | *Morganella morganii* | *Staphylococcus* spp |
| *Chlamydia pneumoniae* | *Mycobacterium avium* | *Stenotrophomonas maltophilia* |
| *Chlamydia psittaci* | *Mycobacterium bovis* | *Streptococcus gordonii* |
| *Chlamydia trachomatis* | *Mycobacterium chelonae* | *Streptococcus mitis/oralis* |
| *Chlamydia trachomatis lgv strain* | *Mycobacterium kansasii* | *Streptococcus pneumoniae* |
| *Citrobacter koseri* | *Mycobacterium tuberculosis* | *Streptococcus viridans* |
| *Clostridium sordellii* | *Mycobacterium xenopi* | *Streptomyces somaliensis* |
| *Corynebacterium spp* | *Mycoplasma fermentans* | *Treponema pallidum* |
| *Coxiella burnetii* | *Mycoplasma genitalium* | *Tsukamurella* spp. |
| *Cutibacterium acnes* | *Mycoplasma hominis* | *Ureaplasma urealyticum* |
|  |  | *Wolbachia pipientis* |

**Detection limit.** To assess sensitivity of the technique, successive dilutions of the *groEl* fragment (1,331 bp) cloned in a puc57 plasmid (2,710 bp) were performed in triplicates. The last concentration for which the three replicates were all amplified corresponded to the detection limit and was converted into copies/µL taking into account that there is only one copy of the *groEl* gene in the genome of *N. mikurensis*. The detection limit was 2.29 copies/µL.

**Table 4.** Detection limit

| **Plasmid concentration** | **copies/µL** | **Replicate 1 (Ct)** | **Replicate 2 (Ct)** | **Replicate 3 (Ct)** |
| --- | --- | --- | --- | --- |
| 1 fg/µL | 229.26 | 30.58 | 30.77 | 30.71 |
| 0.1 fg/µL | 22.92 | 34.09 | 34.01 | 34.05 |
| 10 atg/µL | 2.29 | 36.42 | 36.72 | 36.72 |
| 1 atg/µL | 0.22 | 38.78 | - | - |
| 0.1 atg/µL | 0.02 | - | - | - |
